# Supplementary material for: Content-rich biological network constructed by mining PubMed abstracts
Source: BMC Bioinformatics. 2004 Oct 8;5:147. doi: 10.1186/1471-2105-5-147 (PMC528731; doi:10.1186/1471-2105-5-147)
Supplement: Additional File 2 — The original results of the above study (non-essential files are deleted to keep the file size under the limit set by BMC bioinformatics). [file 1471-2105-5-147-S2.bz2 › chilibotAdditionalFile2/dip10/139ID7739537E497/html/E2F2_TFDP2.html]

 


 **E2F2** and **TFDP2** 
  
Found 8 abstracts in PubMed, retrieved 8.  
 

 What does Google say? 
 PDF only 
| .edu only 

---

**Interactive relationship** (e.g. stimulation, inhibition, etc)

**Neutral relationship**- Both DP 1 and DP 2  [ **TFDP2** ]  synergistically interact with members of the E2F family of proteins, E2F 1, E2F 2  [ **E2F2** ] , and E2F 3, to generate DNA binding complexes that specifically recognize the E2F site and functionally interact with E2F 1 in E2F site dependent transcriptional activation of cellular genes.  Ref: 7865876 Mol Biol Cell, 1994

**Non-interactive relationship** (e.g. studied together, co-existance, homology, etc.)

- No differences were observed between RA sensitive and RA resistant ovarian carcinoma cells in the levels of expression of many cell cycle genes including cyclin A, B and E, cdk and 6, E2F 1, E2F 2  [ **E2F2** ] , E2F 3, E2F 4, E2F 5, DP 1 and DP 2  [ **TFDP2** ] .  Ref: 11753676 Oncogene, 2001
